# Genomic and Functional Characterization of *qnr*-Encoding Plasmids from Municipal Wastewater Biosolid *Klebsiella pneumoniae* Isolates

**DOI:** 10.3389/fmicb.2015.01354

**Published:** 2015-12-08

**Authors:** Ella Kaplan, Noa Sela, Adi Doron-Faigenboim, Shiri Navon-Venezia, Edouard Jurkevitch, Eddie Cytryn

**Affiliations:** ^1^Department of Soil Chemistry, Plant Nutrition and Microbiology, Institute of Soil, Water and Environmental Sciences, The Volcani Center, Agricultural Research OrganizationBeit Dagan, Israel; ^2^Department of Agroecology and Plant Health, The Robert H. Smith Faculty of Agriculture, Food and Environment, The Hebrew University of JerusalemRehovot, Israel; ^3^Department of Plant Pathology, The Volcani Center, Agricultural Research OrganizationBeit Dagan, Israel; ^4^Department of Molecular Biology, Ariel UniversityAriel, Israel

**Keywords:** fluoroquinolone, ciprofloxacin, antibiotic resistance, *qnr*, conjugative plasmids, MIC

## Abstract

Municipal wastewater treatment facilities are considered to be “hotspots” for antibiotic resistance, since they conjoin high densities of environmental and fecal bacteria with selective pressure in the form of sub-therapeutic concentrations of antibiotics. Discharged effluents and biosolids from these facilities can disseminate antibiotic resistant genes to terrestrial and aquatic environments, potentially contributing to the increasing global trend in antibiotic resistance. This phenomenon is especially pertinent when resistance genes are associated with mobile genetic elements such as conjugative plasmids, which can be transferred between bacterial phyla. Fluoroquinolones are among the most abundant antibiotic compounds detected in wastewater treatment facilities, especially in biosolids, where due to their hydrophobic properties they accumulate to concentrations that may exceed 40 mg/L. Although fluoroquinolone resistance is traditionally associated with mutations in the gyrA/topoisomerase IV genes, there is increasing evidence of plasmid-mediated quinolone resistance, which is primarily encoded on *qnr* genes. In this study, we sequenced seven *qnr*-harboring plasmids from a diverse collection of *Klebsiella* strains, isolated from dewatered biosolids from a large wastewater treatment facility in Israel. One of the plasmids, termed pKPSH-11XL was a large (185.4 kbp), multi-drug resistance, IncF-type plasmid that harbored *qnrB* and 10 additional antibiotic resistance genes that conferred resistance to five different antibiotic families. It was highly similar to the pKPN3-like plasmid family that has been detected in multidrug resistant clinical *Klebsiella* isolates. In contrast, the six additional plasmids were much smaller (7–9 Kbp) and harbored a *qnrS* -type gene. These plasmids were highly similar to each other and closely resembled pGNB2, a plasmid isolated from a German wastewater treatment facility. Comparative genome analyses of pKPSH-11XL and other pKPN3-like plasmids concomitant to phylogenetic analysis of housekeeping genes from host *Klebsiella* strains, revealed that these plasmids are limited to a predominantly human-associated sub-clade of *Klebsiella*, suggesting that their host range is very narrow. Conversely, the pGNB2-like plasmids had a much broader host range and appeared to be associated with *Klebsiella* residing in natural environments. This study suggests that: (A) *qnrB-*harboring multidrug-resistant pKPN3-like plasmids can endure the rigorous wastewater treatment process and may therefore be disseminated to downstream environments; and (B) that small *qnrS*-harboring pGNB2-like plasmids are ubiquitous in wastewater treatment facilities and are most likely environmental in origin.

## Introduction

Extensive use and misuse of antibiotics during the past 50 years has led to the rise of resistant bacteria and to the global propagation of antibiotic resistance genes (ARGs; Levy and Marshall, 2004; Jacoby, 2005; Nordmann and Poirel, 2005). ARGs associated with mobile genetic elements (MGEs) capable of self-transferring and dispersion (i.e., phages and conjugative plasmids), are considered to constitute high risk potential to public health (Dröge et al., 2000; Strahilevitz et al., 2007; Smillie et al., 2010; Garcillán-Barcia et al., 2011), since they can be horizontally transmitted across taxonomic boundaries, between phylogenetically diverse groups of bacteria (Wirth et al., 2006; Cattoir et al., 2008; Forsberg et al., 2012; Van Meervenne et al., 2012). In addition to their immediate public health impact (when associated with pathogens), in a broader ecological and epidemiological perspective ARGs have been characterized as “contaminants of emerging concern” (Pruden et al., 2006, 2012) since they can be disseminated from anthropogenic sources into microbiomes in natural environments (Dröge et al., 2000).

Municipal wastewater biosolids, contain approximately 10^12^ bacteria per gram (dry weight), which sustain a prolonged and close proximity within flocs and biofilm. These biofilms encompass both native and fecal-derived bacteria, which often contain mobile elements that harbor ARGs (Parsley et al., 2010; Rahube and Yost, 2010). Furthermore, biosolids often contain antibiotic residues, especially hydrophobic compounds such as fluoroquinolones (Pellegrini et al., 2011; Huerta et al., 2013; Perry and Wright, 2013), which may exert selective pressure on the microbiome. As a consequence, nutrient-rich municipal biosolids, are considered as “hot spots” for horizontal transfer of genetic material (Dröge et al., 2000; Kaplan et al., 2013; Perry and Wright, 2013). The Dan Region Wastewater Treatment facility (the “Shafdan”) is the largest in Israel, treating municipal sewage from the entire Tel Aviv metropolitan region. This includes municipal sewage from approximately 2 million people including four major tertiary hospitals and several clinics. It is therefore an excellent study site for assessing the dynamics of plasmids in wastewater treatment plants. We chose to specifically target plasmids associated with fluoroquinolone resistance given the ubiquitous detection of sub-therapeutic concentrations of this antibiotic in wastewater biosolids (Targeted National Sewage sludge Survey Statistical Analysis Report- http://water.epa.gov/scitech/wastetech/biosolids/tnsss-overview.cfm).

Fluoroquinolones are one of the most commonly prescribed groups of broad spectrum antibacterial drugs. Although initially, resistance to fluoroquinolones was relatively rare and was limited to a number of Gram positive bacteria, since the 1990s resistance has increased rapidly in both Gram-positive and Gram-negative strains (Dalhoff, 2012). Fluoroquinolone resistance was first associated with chromosomal mutations in the bacterial gyrase/topoisomerase IV genes, known as Quinolone-Resistance Determining Regions (QRDRs; Gay et al., 2006). However, it was later demonstrated that resistance can also stem from eﬄux pumps and from specific resistance mechanisms carried on MGEs, such as integrons and conjugative plasmids (Martínez-Martínez et al., 1998; Hata et al., 2005; Strahilevitz et al., 2009; Rodríguez-Martínez et al., 2011; Hua et al., 2014). The *qnr* (quinolone resistance) genes that encode for pentapeptide repeat proteins are considered to be one of the main modes of plasmid-mediated quinolone resistance (PMQR). These elements bind to the bacterial gyrase/topoisomerase IV and thereby hinder quinolone binding (Martínez-Martínez et al., 1998; Tran and Jacoby, 2002; Tran et al., 2005; Strahilevitz et al., 2009). Due to the nature of Qnr-gyrase/topoisomerase IV interactions, they generally confer reduced susceptibility to fluoroquinolones, with MIC levels that are approximately one order of magnitude less than those observed in gyrase/topoisomerase IV mutants (Rodríguez-Martínez et al., 2011). Nonetheless, the clinical importance of these genes is great, due to the fact that they are often associated with multidrug resistance plasmids, and to the capacity of these strains to acquire addition mobile resistance elements that collectively confer clinically relevant resistance (Strahilevitz et al., 2009).

In a previous study (Kaplan et al., 2013), we found that 75% of municipal biosolid *Enterobacteriaceae* isolates that showed reduced susceptibility to ciprofloxacin, (MIC-0.4 μg/ml) harbored at least one *qnr* variant (A, B, or S). Furthermore, we demonstrated that in *Enterobacteriaceae* isolated from dewatered biosolids the level of multi-drug resistance was higher than that observed in isolates from raw sewage, suggesting that activated sludge may select for both multidrug resistant bacteria and PMQR (Munir et al., 2011; Kaplan et al., 2013). The aim of this study was to comprehensively assess the genetic composition of *qnr*-associated plasmids from municipal sewage biosolid isolates, with the overall objective of understanding their epidemiological potential in downstream environments.

## Materials and Methods

### Description of Municipal Biosolid Isolates

The 87 *Enterobacteriaceae* strains used in this study were isolated from municipal biosolids from the “Shafdan” Dan Region Wastewater Treatment and Reclamation Project site, as previously described by Kaplan et al. (2013). All isolates were resistant to 0.4 μg/ml ciprofloxacin, an “intermediate” resistance level that is associated with PMQR (Tran and Jacoby, 2002). This ciprofloxacin concentration was used in order to target isolates harboring PMQRs that could be overlooked with higher concentrations of antibiotics. Fifty of these isolates (57%) characterized as *Klebsiella pneumoniae* by Vitek2 (BioMérieux, Hazelwood, MO, USA), were subsequently given the prefix KPSH (an acronym for *Klebsiella pneumoniae* SHafdan).

### Plasmid Extraction and Purification

Plasmids were extracted as described previously (Brown Kav et al., 2013) with slight modifications as follows: Each isolate was inoculated in 50 ml of Luria-Broth medium with 0.2 μg/ml of ciprofloxacin and incubated overnight at 37°C. Cells were harvested by centrifugation and suspended in 2 ml of solution I (50 mM Glucose, 10 μg/ml RNAse A, 25 mM Tris-Cl pH8, 10 mM EDTA pH8). After suspension, 4 ml of solution II was added (1% sodium dodecyl sulfate, 0.2M NaOH). Tubes were then shaken gently for 20 s, supplemented with 3 ml of solution III (3M Potassium acetate pH4.8) and incubated for 5 min on ice. After incubation tubes were centrifuged at 15,000 × *g* for 10 min. Suspensions were transferred into new tubes and an equal volume of PCI (phenol–chloroform–isoamyl alcohol [25:24:1]) was added and mixed well, and plasmid DNA was collected by centrifugation. Excess phenol was removed by additional centrifugation with pure chloroform. Further ethanol precipitation was conducted by washing the DNA twice with 500 μl of 70% EtOH. Plasmid DNA was separated by horizontal electrophoresis in a 0.8% agarose gel in Tris-acetate-EDTA (TAE) buffer at room temperature at 100 V for 4 h. The molecular mass of the plasmid DNA was assessed by comparison with the migration of plasmids with known molecular masses and to a super-coiled DNA ladder (Supercoiled DNA Marker, Cat. No. SCD31050, Epicentre Biotechnologies Inc., Madison, WI, USA).

### Linear DNA Digestion

Further purification of plasmid DNA was achieved by applying the plasmid-safe DNase digestion kit (Epicentre Biotechnologies Inc., Madison, WI, USA) according to the protocol provided by the manufacturer, which digests linear dsDNA, but not circular (plasmid) DNA.

### Transformation and Antibiotic Susceptibility Profiling of Extracted *Klebsiella pneumoniae* Plasmids

The 49 extracted plasmids were electro transformed into competent DH10B *Escherichia coli* cells using a MicroPulser^TM^ Electroporator (Bio-Rad, cat. #165-2100, Singapore), shaken in 1 ml LB for two hours at 37°C and plated onto LB-agar containing 0.2 μg/ml ciprofloxacin. Plates were incubated for 36–48 h at 37°C. In tandem, non-transformed DH10B *E. coli* cells were plated on identical LB-agar pates to confirm that these competent cells are sensitive to 0.2 μg/ml ciprofloxacin, thereby demonstrating that growth was facilitated by plasmid acquisition. Seven plasmids from *Klebsiella* strains were successfully transformed into the competent *E. coli* strains and served as the core for the rest of this study. The seven electro transformants were screened for resistance against five additional antibiotics using the Clinical and Laboratory Standards Institute guidelines (CLSI) clinical MIC breakpoint concentrations according to standard procedures: Ampicillin 100 μg/ml, Ceftriaxone 2 μg/ml, Tetracycline 30 μg/ml, Chloramphenicol 170 μg/ml, and Nalidixic Acid 32 μg/ml. In addition, the minimal inhibition concentration (MIC) of selected clinically relevant antibiotics [Nalidixic Acid (NX), Ciprofloxacin (CIP), Ceftriaxone (CTX), Ertapenem (ERT), Amikacin (AMK), and Tigecycline (TIG)] was determined for the seven *Klebsiella* isolates using the *E* test method (BioMérieux, Marcy-l’Étoile, France) according to manufacturer’s recommendations. MICs were interpreted according to the updated standards for susceptibility testing, and clinical MIC breakpoints from the Clinical and Laboratory Standards Institute guidelines (CLSI 2012, 2013).

### Sequencing and Assembly of Selected Quinolone-resistant Plasmids

Plasmids from the seven *E. coli* DH10β transformants were extracted as described above. Removal of chromosomal DNA was validated by PCR targeting of the 16S rRNA gene as previously described (Marilley et al., 1998). One nanogram of plasmid DNA from each transformant was fragmented, tagged with adapters, and libraries were prepared using the Nextera XT DNA Sample Preparation Kit according to the manufacturer’s protocol (Nextera XT, Illumina Inc., San Diego 92122, CA, USA). Samples were then sequenced using the Illumina MiSeq platform (Illumina, San Diego, CA, USA).

Adaptor sequences were removed from the raw sequence reads and low quality sequences were removed using Trimmomatic version 0.32 (Bolger et al., 2014). Reads were then assembled into large contigs using the a5 assembler pipeline (Tritt et al., 2012), and whole plasmids were closed by genome reference assisted scaffolding using the CAR software tool (Lu et al., 2014). Rapid Annotation of plasmids was achieved using Subsystem Technology (RAST) software version 2.0 (Aziz et al., 2008) and ARGs were annotated using the Comprehensive Antibiotic Resistance Database (CARD) package (McArthur et al., 2013). Comparative plasmid maps were generated from the assembled contigs using the BLAST Ring Image Generator (BRIG) software version 0.95 (Alikhan et al., 2011).

### Phylogenetic Association of Plasmid-Harboring *Klebsiella* Host Strains

Phylogenetic characterization of plasmid-harboring *Klebsiella* host strains was initially conducted by Multi Locus Sequence Typing (MLST), using protocols developed by the Pasteur Institute^1^. However, some of these housekeeping genes, such as *mdh*, *pgi*, *phoE*, *infB*, and *tonB* failed to generate PCR products in some of the sludge *Klebsiella* isolates. We therefore specifically focused on *gapA* and *rpoB* (encoding for glyceraldehyde 3-phosphate dehydrogenase and beta-subunit of RNA polymerase, respectively) for phylogenetic characterization of the strains. DNA was extracted from the *Klebsiella* isolates as previously described (Kaplan et al., 2013), and *gapA* and *rpoB* genes were amplified by PCR using the Pasteur Institute primers. PCR products were visualized by 1% agarose gel electrophoresis, and were sequenced using the gapA173F (gapA) and Vic3 (rpoB) primers detailed on the Pasteur website^2^ using standard Sanger sequencing.

G*apA* and *rpoB* sequences were aligned with MUSCLE^3^ using default parameters. Phylogenetic trees of individual gene alignments and of a concatenated *gapA* and *rpoB* alignment were constructed using maximum likelihood (ML) with PhyML software version 3.0 based on a GTR model with 100 bootstrap repeats (Guindon et al., 2010). The tree was graphically constructed using FigTree 1.4.2^4^.

### Plasmid Sequence Accession Numbers

Plasmid sequences were deposited into the NCBI database under accession numbers KT896499 to KT896504 (Supplementary Data Sheets 1–6).

## Results

### Antibiotic Screening of Plasmid-transformed *E. coli* Recipients

Of the 87 isolates screened, 49 contained one or more plasmids, and seven of these plasmids were successfully electro transformed into naïve *E. coli* DH10B cells. The sizes of these plasmids were estimated to range between 8 to 185 kbp. The native KPSH strains and the electro transformants were screened against five different antibiotics on Luria–Bertani (LB) agar as shown in **Table 1**. Ciprofloxacin resistance was evaluated based on plasmid (subMIC) and chromosome-associated MICs (0.2 μg/ml and 4 μg/ml, respectively) according to Tran and Jacoby (2002) who demonstrated that PMQR is approximately 10-fold lower than traditional gyrase/topoisomerase IV mutation-associated MIC’s.

**Table 1:** Antibiotic resistance of the seven “donor” *Klebsiella* isolates and their respective DH10β transformants.

Isolate strain	Isolation date	Resistances of *Klebsiella* isolates	Resistances of recipient *Escherichia coli* DH_10_β cells
KPSH-11XL	13.7.2011	Ciprofloxacin, Ampicillin, Ceftriaxone, Tetracycline, Chloramphenicol	Ciprofloxacin (subMIC), Ampicillin, Ceftriaxone, Tetracycline
KPSH-70	14.11. 2011	Ciprofloxacin, Ampicillin, Chloramphenicol, Nalidixic acid	Ciprofloxacin (subMIC)
KPSH-169	30.4. 2012	Ciprofloxacin, Ampicillin, Ceftriaxone, Chloramphenicol	Ciprofloxacin. (subMIC)
KPSH-201	28.5. 2012	Ciprofloxacin (subMIC)	Ciprofloxacin (subMIC)
KPSH-212	28.5. 2012	Ciprofloxacin, Ampicillin, Nalidixic acid	Ciprofloxacin (subMIC)
KPSH-213	28.5. 2012	Ciprofloxacin	Ciprofloxacin (subMIC)
KPSH-231	10.10. 2012	Ciprofloxacin, Ampicillin, Nalidixic acid	Ciprofloxacin (subMIC)


*Concentrations of antibiotics used: Ampicillin 100 μg/ml, Ceftriaxone 2 μg/ml, Tetracycline 30 μg/ml, Chloramphenicol 170 μg/ml, ciprofloxacin 4 μg/ml, subMIC ciprofloxacin – 0.2 μg/ml.*

Plasmid KPSH-11XL conferred resistance to four of the screened antibiotics, whereas the other six plasmids only conferred resistance to sub-MIC levels of ciprofloxacin. Further investigation of these plasmids using *E* test (BioMérieux, France) enabled a more robust assessment of MIC levels of native and plasmid-transformed *E. coli* DH10B cells. Analyses revealed that the transformants acquired different levels of resistance to several of the analyzed antibiotics (**Table 2**). Two plasmids that were extracted from the KPSH-213 strain, displayed very different MICs toward the five antibiotic compounds tested, as depicted in **Table 2**. These two plasmids are termed pKPSH-213.1 and pKPSH-213.55.

**Table 2:** MIC levels (in μg/ml) of *E. coli* DH10β transformants and non-transformed recipients.

	NX	CIP	CTX	ERT	AMK	TIG
Recipient DH10β	1	<0.002	0.064	0.006	2	0.047
pKPSH-11XL	1.5	0.125	**32**	0.012	3	0.094
pKPSH-70	8	0.25	0.19	0.023	1.5	0.125
pKPSH-169	4	0.25	0.094	0.008	1.5	0.064
pKPSH-201	4	0.19	0.064	0.008	1.5	0.094
pKPSH-213.1	4	0.25	0.094	0.006	1	0.032
pKPSH-213.55	32	0.5	0.125	0.006	1.5	0.125
pKPSH-231	3	0.125	0.094	0.006	2	0.047


*The Minimum inhibition concentration (MIC) of the following antibiotics were measured using the *E* test method (BioMérieux, Marcy-l’Étoile, France) according to manufacturer’s recommendations: Nalidixic Acid (NX), Ciprofloxacin (CIP), Ceftriaxone (CTX), Ertapenem (ERT), Amikacin (AMK), and Tigecycline (TIG). Only ceftriaxone resistance in plasmid pKPSH-11XL (in bold) exceeded CLSI MIC values for *Enterobacteriaceae* (updated standards M02-A11 and M07-A9).*

### Sequencing and Annotation of *qnr*-Associated Plasmids

**FIGURE 1:**
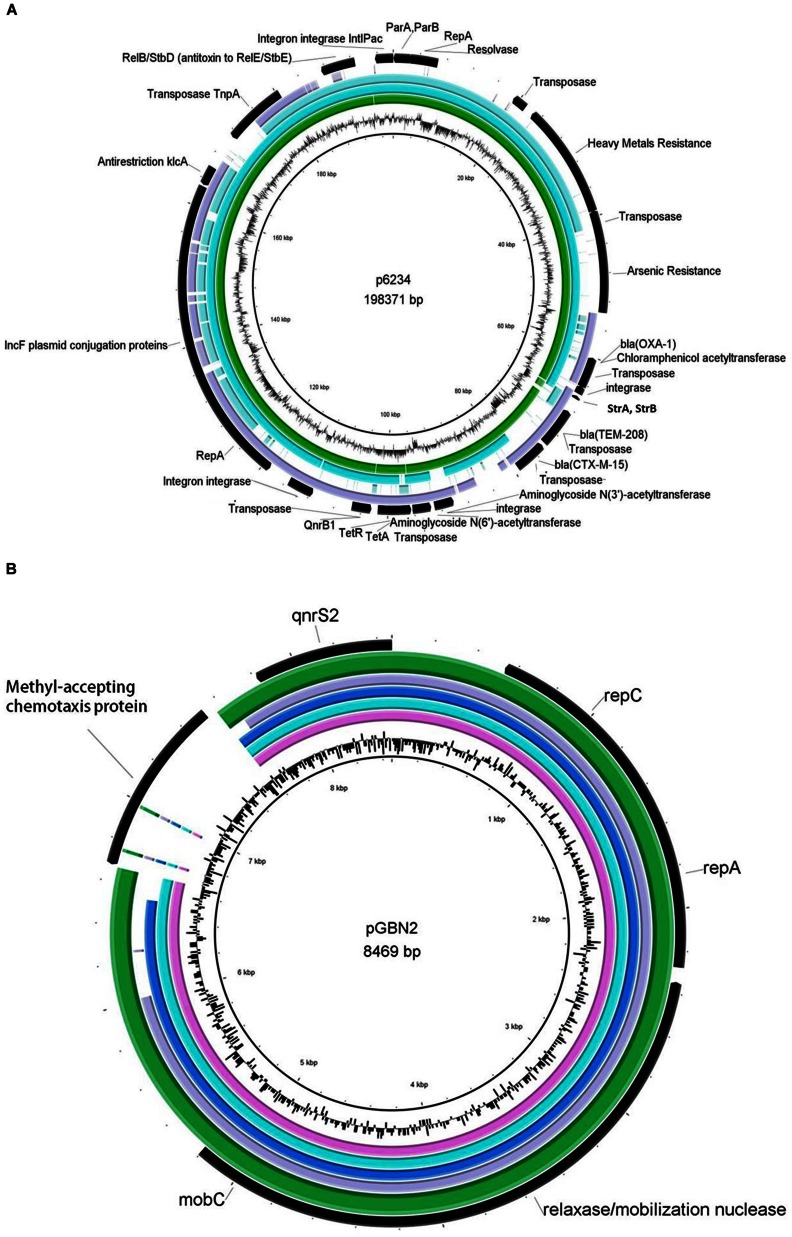
**Genetic maps of sequenced plasmids aligned with closely related reference strains.** **(A)** pKPSH-11XL (green ring), pKPN-c22 (turquoise ring) pKPN_CZ (light blue ring), and pKDO1 (purple ring) are aligned to reference sequence p6234, which showed the highest resemblance to pKPSH11-XL (middle black ring). Annotations of pKPSH-11XL encoded proteins appear in the outer black ring, and GC content of the reference plasmid p6234 is displayed between the inner black and green rings. **(B)** The six pGNB2-like plasmids: pKPSH-70 (pink ring), pKPSH-169 (turquoise ring), pKPSH-212 (blue ring), pKPSH-213.55 (purple ring), and pKPSH-231 (green ring), aligned to the reference pGNB2 sequence (middle black ring). Annotations of relevant encoded proteins from the six sequenced plasmids appear in the outer black ring, whereas GC content of reference plasmid is displayed between the inner black and green rings. Figures were generated with the BRIG software package using default parameters.

Sequencing of the seven plasmids from the ciprofloxacin resistant *Klebsiella* isolates revealed two types of plasmids (**Figure 1**). A 185.4 Kbp Inc-F plasmid termed pKPSH-11XL, which harbored *qnrB*, three types of beta-lactamase genes (*bla*_OXA-1_, *bla*_CTXM-15_, and *bla*_TEM-208_), tetracycline resistance gene *tetA* (and its regulator, *tetR*), two aminoglycoside 3′-phosphotransferases (*strA* and *strB* conferring resistance to streptomycin), one aminoglycoside N(6′)-acetyltransferase (known to confer resistance to both aminoglycosides and fluoroquinolones), one aminoglycoside N(3′)-acetyltransferase and one chloramphenicol acetyltransferase (**Figure 1**). This mega plasmid also contained a cluster of six genes conferring arsenic resistance, 10 genes encoding for copper, lead, cadmium, zinc, and mercury resistance as well as several genes encoding mobilization elements including transposases, integrases, a recombinase, an anti-restriction mechanism (*klcA*) and a RelB/StbD toxin-antitoxin addiction apparatus. In addition, it encoded a cluster of 25 IncF plasmid conjugation genes. Collectively, these data indicated that pKPSH-11XL was a multidrug-resistance conjugative plasmid belonging to the previously described *K. pneumoniae*-associated pKPN3-like plasmid family.

Multiple sequence alignment and comparative genomics of pKPSH-11XL with five of the most similar *K. pneumonia*-associated pKPN3-like plasmids is shown in **Figure 1** and **Table 3**, respectively. These plasmids were characterized by a distinct structure, with spatial separation between an adaptive module (that contained all of the resistance genes) and a backbone which contained all of the genes encoding for conjugation, replication, stability, and partitioning of the plasmid. Although highly similar, comparative genomic analysis revealed evidence of some gene loss and acquisition between these different plasmids (**Table 3**).

**Table 3:** Comparison of adaptive module genes in pKPSH-11XL and other IncF pKPN3-like plasmids.

	pKPSH-11XL	P6234	pKPN-c22	pKDO1	pKPQIL	pKPN_CZ	pKPN3
Accession number	KT896504	CP010390.1	CP009879.1	NC019389.1	NC014016.1	JX424424.1	NC009649.1
*Klebsiella pneumoniae* parent strain	KPSH-11XL	subsp. 6234	subsp. NIH31	clone ST416	subsp. ST-258	clone ST416	subsp. MGH78578
Plasmid size	185,423 bp	198,371 bp	183,785 bp	131,207 bp	115,349 bp	210,875 bp	178,507 bp
Plasmid partitioning proteins ParA and ParB^∗^	+	+	+		+	+	+
RelB/StbD replicon stabilization	+	+	+			+	+
Arsenic resistance cluster	+	+	+				+
Heavy metal resistance cluster	+	+	+			+	+
Integron integrase IntIPac	+	+	+	+	+		
Aminoglycoside N(6′)-acetyltransferase	+	+	+	+			
Aminoglycoside 3′-phosphotransferase- StrA and StrB	+	+		+			
Beta-Lactamase CTX-M-15^∗∗^	+	+		+	+		
Beta-Lactamase TEM-208^∗∗∗^	+	+		+	+		
Beta-Lactamase OXA-1^∗∗^	+	+	+	+	+		
Chloramphenicol acetyltransferase	+						
Tetracycline eﬄux TetA and Tet R	+	+	+	+			
Fluoroquinolone resistance QnrB	+	+	+	+			
Isolation source	Municipal biosolids- Israel	Body fluid, Colombia	Hematologic malignancy, USA	Oncological clinic, Czech republic	*K. pneumoniae* outbreak- Israel	Oncological clinic, Czech republic	Blood sample, USA
Reference	This study	Unpublished	Conlan et al., 2014	Dolejska et al., 2013	Leavitt et al., 2010	Dolejska et al., 2013	*K. pneumoniae* genome sequencing project


*^∗^pKDO1 encodes for only ParA; ^∗∗^pKPQIL encodes for KPC-3 and not for CTX-M-15, and for OXA-9 (77% similarity to OXA-1); ^∗∗∗^pKPSH-11XL encodes for TEM-208 (99% similarity toTEM-1).*

The six additional plasmids (pKPSH-70, pKPSH-169, pKPSH-212, pKPSH-213.1, pKPSH-213.55, and pKPSH-231) were much smaller than pKPSH-11XL. Their average size was 8.5 Kbp and they all contained the plasmid mediated quinolone resistance determinant *qnrS*, the plasmid replication initiator *repC*, and a Relaxasase/Mobilization nuclease domain. Additionally, five of the six plasmids (excluding pKPSH-213.55) encoded the Helicase RepA, and four of the plasmids (pKPSH 70,169,212,231) also encoded the mobilization protein MobC (**Figure 1**). The *qnrS* gene was 100% identical in all six plasmids, and to the *qnrS* harbored on both the IncQ-family plasmid pGNB2 isolated by plasmid capture from a wastewater treatment plant in Germany (Bönemann et al., 2006) and the IncU plasmid pBRST7.6 isolated from an *Aeromonas hydrophila* fish pathogen (Majumdar et al., 2011). *In silico* examination of the replicase gene sequence of these plasmids using PlasmidFinder 1.3 software (Carattoli et al., 2014) confirmed that they were all IncQ2 type plasmids.

### Phylogenetic Association of *qnr* Plasmid-harboring *Klebsiella* Host Strains

**FIGURE 2:**
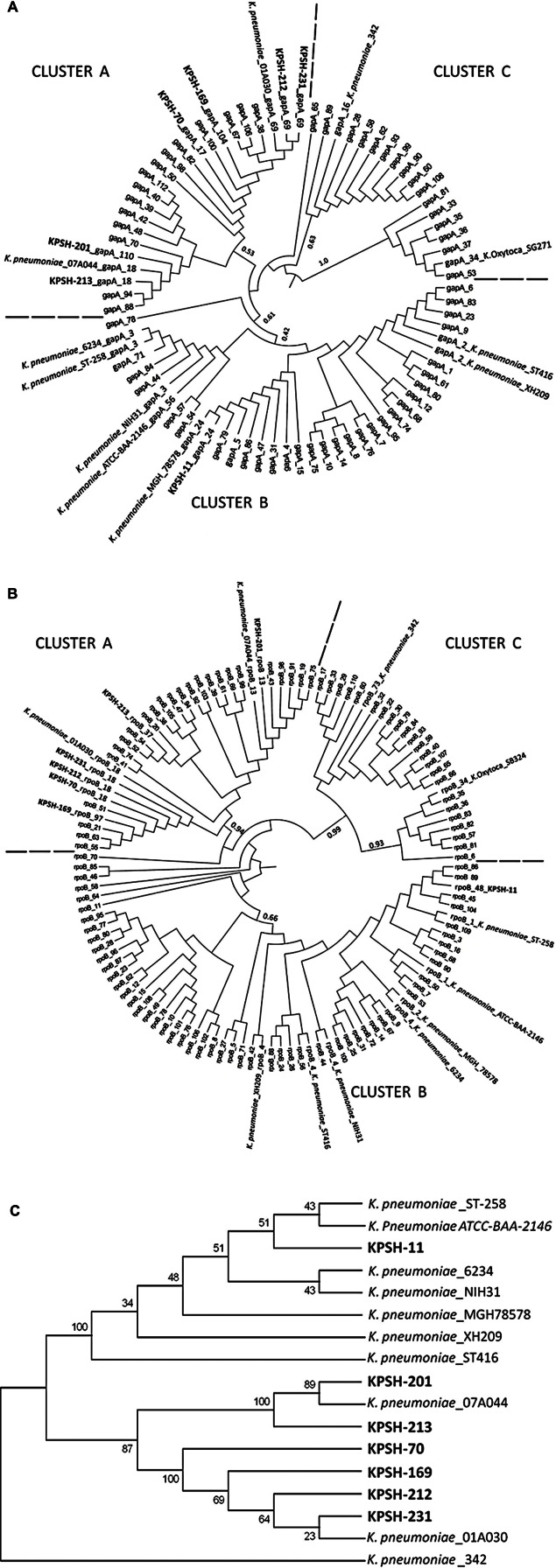
**Phylogenetic affiliation of *Klebsiella pneumoniae* isolates from this study (in bold) relative to reference strains.** Comprehensive trees constructed using *gapA* **(A)**, and *rpoB* **(B)** housekeeping gene sequences. Condensed concatenated tree based on both *gapA* and *rpoB* genes specifically depicting host strains from this study **(C)**. The tree was rooted with the wheat root isolate *K. pneumoniae* strain 342 (Fouts et al., 2008). The seven KPSH isolates segregated into two phylogenetically distinct clusters: Cluster A included the six pGBN2-like plasmids, which segregated into two sub-clusters. The first encompassed strains KPSH-201 and KPSH-213 and *K. pneumoniae* strain 07A044 isolated from a blood culture of a patient in Freiburg, Germany (Brisse et al., 2014), while the second contained KPSH-70, KPSH-169, KPSH-212, and KPSH-231 and *K. pneumoniae* strain 01A030 isolated from a blood culture of a patient in Linz, Austria (Brisse et al., 2014). Cluster B contained KPSH-11 and all of the pKPN3-like harboring *K. pneumoniae* strains. It was most closely related to ATCC-BAA-2146 that was isolated from the urine of a U.S. hospital patient (Hudson et al., 2014), and ST-258, a globally disseminated extremely drug-resistant KPC-3-producing strain isolated also in Israel, that harbored the pKpQIL plasmid (Leavitt et al., 2010) described in **Table 3**. Other depicted *K. pneumoniae* strains include 6234- harbors p6234 which is highly resemble to pKPSH-11XL, isolated from bodily fluid of a patient from Colombia, ST-416- harbors both pKDO1 and pKPN-CZ, isolated from a pediatric oncological clinic in the Czech republic (Dolejska et al., 2013); XH209- A multidrug-resistant strain isolated from the blood of a patient in Hangzhou, Zhejiang, China (Hua et al., 2014); and 78578: Multi resistant clinical strain isolated from sputum from Japan (Ogawa et al., 2005).

The highly conserved housekeeping genes *gapA* and *rpoB* (encoding for the bacterial glyceraldehyde 3-phosphate dehydrogenase and for the beta-subunit of RNA polymerase, respectively) of the *K. pneumoniae* isolates described in this study and from an array of reference strains from the Pasteur institute MLST database^5^ were used to construct phylogenetic trees (**Figures 2A,B**, respectively). A more concise concatenated tree based on the aligned sequences of both genes was then constructed (**Figure 2C**) in order to assess the phylogenetic relation of the plasmid-harboring isolates relative to closely-related clinical and environmental *Klebsiella* strains.

Trees were constructed based on the GTR model with 1000 bootstrap repeats, of all the available alleles from the Pasteur MLST database.

## Discussion

Understanding mechanisms associated with dissemination of mobile ARGs in environmental microbiomes is crucial to public health, given the impact they have on the global propagation of antibiotic resistance. This is especially relevant in the case of broad host range plasmids, because they can traverse phylogenetic and environmental barriers. Plasmids harboring ARGs have been well defined in bacterial pathogens isolated from clinical settings and their epidemiological contribution is well understood. Conversely, there is much less knowledge regarding ARG-harboring plasmids in non-clinical environments. Our work focused on plasmids from *K. pneumoniae* strains isolated from municipal wastewater biosolids, a unique niche in which high concentrations of fecal and environmental bacteria coexist under a steady selective force, where conjugation can readily occur within the close proximity of condensed biofilms. We therefore elected to characterize potentially conjugative plasmids from this environment, to acquire a deeper understanding of the genomic mechanisms associated with these plasmids. *K. pneumoniae* was chosen as a model species in this study due to its high abundance in municipal biosolids (Kaplan et al., 2013), its clinical relevance and the fact that it has been linked to horizontal transfer of *qnr*-encoding plasmids (Wang et al., 2004).

In this study, we identified two very different types of quinolone resistance gene-encoding plasmids, in *K. pneumoniae* strains isolated from dewatered biosolids. The first (pKPSH-11XL) was a large (185.4 kbp) multidrug-resistant, IncF plasmid closely related to members of the previously described pKP3 family, which has been frequently detected in clinical settings. These plasmids often confer multi-drug resistance and have been linked to the dissemination of ARGs such as *bla*_CTX-M_, *qnr*, and aminoglycoside resistance genes (Villa et al., 2010). This finding correlates to previous reports that determined that plasmids that confer multi-drug resistance are usually large (>50 kb), and self-conjugative (Carattoli, 2013). The exact origin of pKPSH-11XL is unknown, but to the best of our knowledge this is the first documentation of such a plasmid in a host-free environment. The capacity of this plasmid to persist and be mobilized in natural environments is currently unknown, but the fact that it endured the exhaustive activated sludge process indicates that it is rather robust. Comparative genomics of pKPSH-11XL and closely related pKPN3 plasmids from clinical isolates was conducted to better understand the evolution of this plasmid family and evaluate whether it contains unique elements that may indicate its capacity to persist in natural environments. Evaluation of the adaptive modules as well as the replication, stabilization, partitioning, and conjugation modules of these plasmids (**Table 3**) clearly indicated that although similar, the plasmids are not identical. We detected differences in presence of aminoglycoside and chloramphenicol resistance genes, and slightly different composition of arsenic and heavy metal resistance gene clusters (**Table 3**). These differences may stem from different selection pressures that promoted acquisition (or loss) of specific genetic elements, or conversely, co-evolution of an ancestral plasmid prototype. Phylogenetic analysis of the *Klebsiella* host strains based on *gapA* and *rpoB* gene sequences (**Figure 2**) revealed that strains harboring pKPN3-like plasmids (including KPSH-11) were closely related to each other despite their geographical disparity (Ogawa et al., 2005; Conlan et al., 2014). This cladding suggests that pKPN3-like plasmids have a very narrow host range. However, the pKPSH-11XL efficiently conferred antibiotic resistance when transformed and conjugated (data not shown) into competent DH10B *E. coli* cells, suggesting that this limitation stems from a selective boundary and not a mechanistic one. We are currently conducting experiments aimed to assess transferability rates and stability of this plasmid in various *Klebsiella* and related *Enterobacteriaceae* strains. Plasmids like pKPN3 that encode for anti-restriction, partitioning and addiction mechanisms, are generally considered to be promiscuous (Villa et al., 2010; Carattoli, 2013). However, the fact that all of the characterized strains hosting these plasmids are associated with a specific cluster within the *Klebsiella* genus indicates that de-facto they may be restricted to a relatively narrow host range (**Figure 2**).

The six pGNB-2 like plasmid harboring *K. pneumoniae* strains formed a completely separate clade, which were most closely related to the chromosomal encoding beta-lactamase strain 07A044 (Brisse et al., 2014). Again, all of the host strains were phylogenetically related, despite the fact that they were isolated from biosolids sampled at four different time points during different seasons (November 2011 and April, May, and October 2012). The association between *Klebsiella* strain phylogeny and plasmid type could indicate co-evolution or preference of specific host types to specific plasmid types in this environment. All of these plasmids contained a *qnrS* gene that was100% identical to *qnrS* encoded on both the IncQ-family pGNB2 isolated from a German WWTP (Bönemann et al., 2006); and the IncU pBRST7.6 isolated from an *Aeromonas hydrophila* fish pathogen (Majumdar et al., 2011). The abundance of *qnrS* relative to other *qnr* variants (∼44%) in our biosolid *K. pneumoniae* collection (Kaplan et al., 2013), is significantly higher than in clinical environments in Israel and around the world, where the abundance of *qnrS* variants is generally below 10% (Silva-Sánchez et al., 2013; Strahilevitz personal comunication (Cattoir et al., 2007; Jiang et al., 2008; Tripathi et al., 2012; Azadpour et al., 2014). High prevalence of *qnrS* has also detected in WWTP *Aeromonas* isolates in Portugal, contrary to low levels of *qnrS* in clinical isolates there (Prof. Celia Manaia, personal communication), supporting the notion that *qnrS* may confer a selective advantage to bacteria in WWTPs, and indicating a broader phylogenetic and ecological distribution of this plasmid group. In addition to identification in metagenomes of other wastewater samples, we recently also identified *qnrS* in soil metagenomes with no evident anthropogenic influence (Gatica et al., unpublished). This suggests that these genes might have a broader role in the environment, beyond resistance to fluoroquinolones.

The German WWTP plasmid pGNB2 contained a methyl-accepting chemotaxis protein (MCP, 96% identical to an *Aeromonas* protein, accession number: WP_053288181.1) that was absent in all six of the plasmids analyzed in this study. MCPs are associated with rotor activity of the bacterial flagella and are believed to be associated with chemotaxis toward specific attractants (Derr et al., 2006). The lack of MCP on this plasmid may stem from the fact that *Klebsiella* do not possess flagella. Interestingly, this suggests that these plasmids may be subjected to “editing” based on the physiological needs of the bacterial host.

As previously discussed, PMQR generally confers resistance to fluoroquinolones at sub-MIC concentrations of up to 0.5 μg/ml, which is about one tenth of the resistance obtained by mutation in the bacterial gyrase/topoisomerase genes (Tran and Jacoby, 2002). However, the MICs for ciprofloxacin of most of the host bacteria harboring the GNB2-like plasmids were much higher than those of the plasmid-transformed *E. coli* DH10B competent cells (**Table 1**). This again raises a very important question regarding the selective advantage these plasmids confer to the host strains. Presumably, harboring *qnrS* contributes little, if any, to the cells’ ability to survive selective pressure caused by ciprofloxacin exposure, so other benefits must come from maintaining these GNB2-like plasmids in it. These might include bacterial metabolic processes, communication, and signaling. Alternatively, they may provide an additive effect where the presence of a *qnr* allow the accumulations of mutations in the bacterial gyrase and topoisomerase IV (Martinez, 2009; Rodríguez-Martínez et al., 2011).

Ciprofloxacin MIC values of the transformed DH10B strains shown in **Table 2** were at least 62.5–250 times higher than the native DH10B strains, indicating that they significantly contribute to ciprofloxacin resistance, although still below clinical resistance level. The differences in MIC levels measured in different electro transformants carrying the same *qnrS* gene (shown in **Table 2**) may be attributed to different factors such as plasmid copy number, gene regulation, and expression, which may be different in the *E. coli* DH10B recipient relative to the host *Klebsiella* strains.

The complete genome of the *K. pneumoniae* strain KPSH-11was recently sequenced (data not shown). Interestingly, the QRDRs, i.e., the two subunits of both the bacterial gyrase and topoisomerase IV (gyrA, gyrB, parC, and parE, respectively) did not contain characterized mutations known to confer quinolone resistance and therefore the mechanism that is responsible for conferring the high (4 μg/ml) MIC values observed in this strain are currently an enigma.

Collectively, these findings indicate that fluoroquinolone resistance in environmental *K. pneumoniae* strains may be more complex than what is currently known from clinical strains and that *qnr* genes may have additional roles in the environment, beyond conferring resistance to fluoroquinolones. Additional research is required to the capacity of plasmid-associated *qnr* genes in natural soil and water microbiomes.

## Author Contributions

EK designed experiments, conducted research, and wrote manuscript; NS and ADF contributed to genomic and phylogenetic analyses; SNV contributed to strain sequencing and manuscript editing; EJ supervised research; EC supervised research, contributed to experimental design, contributed to writing manuscript.

## Conflict of Interest Statement

The authors declare that the research was conducted in the absence of any commercial or financial relationships that could be construed as a potential conflict of interest.

## Footnotes

1 http://bigsdb.web.pasteur.fr/klebsiella/klebsiella.html

2 http://bigsdb.web.pasteur.fr/klebsiella/primers_used.html

3 www.ebi.ac.uk/Tools/msa/muscle/

4 http://tree.bio.ed.ac.uk/software/figtree

5 bigsdb.web.pasteur.fr/klebsiella/klebsiella.html
